# Clinical utility of the Structured Observation of Motor Performance in Infants within the child health services

**DOI:** 10.1371/journal.pone.0181398

**Published:** 2017-07-19

**Authors:** Kine Johansen, Kristina Persson, Karin Sonnander, Margaretha Magnusson, Anna Sarkadi, Steven Lucas

**Affiliations:** 1 Department of Women’s and Children’s Health, Uppsala University, Uppsala, Sweden; 2 Uppsala University Children’s Hospital, Uppsala, Sweden; 3 Department of Public Health and Caring Sciences, Uppsala University, Uppsala, Sweden; Boston Children's Hospital / Harvard Medical School, UNITED STATES

## Abstract

**Aim:**

This study aimed to evaluate the clinical utility of the Structured Observation of Motor Performance in Infants (SOMP-I) when used by nurses in routine child healthcare by analyzing the nurses’ SOMP-I assessments and the actions taken when motor problems were suspected.

**Method:**

Infants from three child health centers in Uppsala County, Sweden, were consecutively enrolled in a longitudinal study. The 242 infants were assessed using SOMP-I by the nurse responsible for the infant as part of the regular well-child visits at as close to 2, 4, 6 and 10 months of age as possible. The nurses noted actions taken such as giving advice, scheduling an extra follow-up or referring the infant to specialized care. The infants’ motor development was reassessed at 18 months of age through review of medical records or parental report.

**Results:**

The assessments of level of motor development at 2 and 10 months showed a distribution corresponding to the percentile distribution of the SOMP-I method. Fewer infants than expected were assessed as delayed at 4 and 6 months or deficient in quality at all assessment ages. When an infant was assessed as delayed in level or deficient in quality, the likelihood of the nurse taking actions increased. This increased further if both delay and quality deficit were found at the same assessment or if one or both were found at repeated assessments. The reassessment of the motor development at 18 months did not reveal any missed infants with major motor impairments.

**Interpretation:**

The use of SOMP-I appears to demonstrate favorable clinical utility in routine child healthcare as tested here. Child health nurses can assess early motor performance using this standardized assessment method, and using the method appears to support them the clinical decision-making.

## Introduction

Signs of motor delay or aberrant motor performance in infancy may signal the presence of a motor disorder or other developmental problems[1,2]. These signs may be nonspecific,[3] but early detection is crucial to enable interventions that can improve outcomes[2,4–11]. Timely interventions can prevent or minimize developmental delays,[5–8,11–13] as well as prevent unnecessary secondary effects of a motor disorder[6,7,10]. The intervention should commence during the first months of life when the brain is most plastic,[4–9,11,14] and therapy should preferably be initiated before a motor disorder is evident or the infant is delayed in achieving the expected milestones[5–7,11,13,15].

To facilitate early detection of motor problems and developmental delays the use of standardized assessment methods is recommended[2,10,16]. Not only does assessment using standardized methods improve the accuracy of detection, it also provides valuable information compared to clinical judgment alone[16–19]. Health professionals that rely only on their clinical judgment detect fewer children with developmental delays and do so at a later stage compared to professionals that apply a standardized assessment method[2,16,17,20–22]. To improve detection rates during infancy repeated assessments are recommended[23–26].

Assessment methods that are based on scientifically supported theories and that are psychometrically sound should be chosen when available[27,28]. Furthermore, the assessment method should have relevance for the intended purpose and be clinically useful to the practitioner[27–31]. Clinical usefulness, or clinical utility, refers to the ease of using a method, and is concerned with the practical advantage the method offers the practitioner[32–34]. Smart has proposed a model of clinical utility based on four dimensions with the aim of revealing the benefits and drawbacks of a method in a specific setting[35]. *Appropriateness*, refers to whether the method is effective and relevant, while *accessibility* evaluates among others the resource implications of implementing the method. For the assessment method to be *practicable* it has to be compatible with the practitioner’s needs and capabilities. This dimension covers aspects like the functionality of the assessment method and also its suitability in a particular context. Furthermore, to have clinical utility the method has to be *acceptable* to the practitioner, the client and the society.

The Swedish child health services (CHS) are responsible for promoting and monitoring the health and development of all children from birth until school entry (0–6 years)[36]. The CHS are well regarded by parents, and the service has an attendance rate of over 99%[36,37]. The CHS program is comprehensive, with repeated well-child visits that are all voluntary and free of charge. The majority of the visits occur during the child’s first year of life,[38] with the first only days after the infant is discharged from the hospital. As nurses are the hub of the child health center’s daily work, they have a key medical role in the CHS[36,37].

The goals of the Swedish CHS are to promote children’s health and development, to prevent ill-health and to strive for early detection of problems in health, development and/or the environment as well as initiate appropriate actions to target these[36]. This monitoring of development includes being responsive to parental concerns, performing skilled observations of the child, and providing anticipatory guidance on health and developmental issues relevant to the child’s age and the parents’ needs[39,40]. Given the regular contact between the CHS and the families, the nurse is ideally placed to monitor development, detect emerging problems and intervene when needed[39].

Today no standardized assessment methods for motor development are used in routine CHS practice,[36,41] and motor development is instead monitored through milestone attainment. The motor milestones originate from early theories that explain motor development as a pre-set and sequential progress from one skill to another[42,43]. However, contemporary theories imply that motor development is a flexible and adaptive process that is dependent of the continuous feedback between the brain, the body and the environment[44–47]. The observation of milestones attainment does not reflect this complex process of motor development. Furthermore, milestones appear with great variation during the first year of life,[15] and they tell us nothing about what may underlie a delay in achieving them[23]. They also give a rough measure of motor development applied in a binary fashion, which makes early detection of children with motor disorders difficult[15,47,48]. In contrast, assessment of the quality of motor performance, which describes how movements are performed, can enable earlier detection of children with motor disorders,[2,5,23,24,49–51] and thereby avoid the loss of valuable time for intervention that waiting for milestones attainment could entail[5–7,12,13,15,26,50,52].

Today’s assessment of milestones is not regulated as to how the observation should be performed and parental report is considered acceptable[36,41]. Parental concern is considered a significant marker for development disorders,[16,53,54] but this is not necessarily true when it comes to early motor problems. For instance, Ehrmann Feldman and colleagues showed that physicians are concerned significantly earlier than parents of younger children with neuromotor problems[55].

For an assessment method to be clinically useful in the child healthcare setting it must meet a number of requirements. First, it should assess both level, i.e. the progress of development, and quality of motor behavior to increase the possibility of early detection,[2,49] as well as allow repeated assessments to enhance the predictive ability[23–25,56,57]. In addition, the method must be quick and easy to perform in a busy clinical setting,[27,28,30,31,35] as well as cover the range of motor problems that are encountered in the primary healthcare setting and support the nurse in the decision-making process[30,58].

The Structured Observation of Motor Performance in Infants (SOMP-I) assesses early motor performance (0–12 months) through detailed descriptions of level of motor development and quality of motor performance[59,60]. SOMP-I is a non-diagnostic, primarily discriminative method that aims to detect aberrant motor performance that may require intervention regardless of the problem’s severity and etiology[51,61]. SOMP-I is quick and non-invasive, and is suitable for a busy clinical setting when used by physiotherapists. Therefore, we hypothesized that the method could be useful in the child healthcare setting as well.

Previously, we have investigated the method’s reliability and some aspects of clinical utility when used by nurses in the child healthcare setting[62,63]. Despite having only brief training and experience with SOMP-I, nurses were able to reliably assess the level of motor development, while more variability was found when assessing the quality of motor performance[63]. In focus group interviews, the child health nurses expressed that learning the method was relevant and provided them with a tool to master a central task in their daily clinical practice[62].

To further elucidate the clinical utility of SOMP-I in the child healthcare setting, the aim of this study was to evaluate the appropriateness, practicability and acceptability of the method when used by child health nurses in routine care. We describe the infants’ motor performance when assessed by nurses using SOMP-I at single and repeated assessments, as well as actions taken by the nurses in regards to motor performance in routine practice. To ensure that no infants with major motor impairments were missed, we investigated the infants’ motor development at 18 months of age.

## Methods

### Participants and procedure

**Fig 1:**
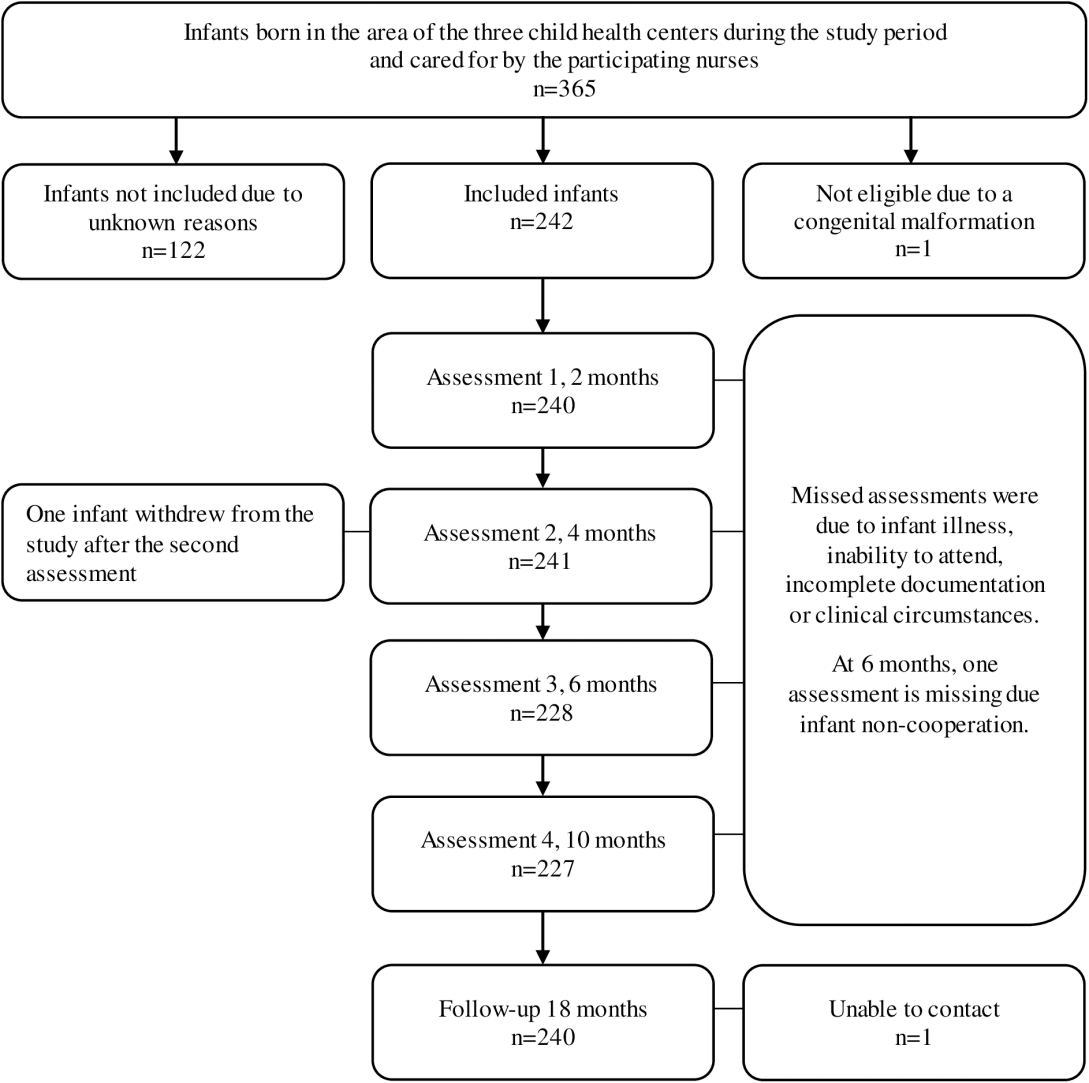
Flow-chart of recruitment and follow-up assessments.

Between March 2013 and February 2014, 243 infants (124 girls) from three child health centers in Uppsala County, Sweden, were consecutively enrolled in a longitudinal study. This represented 67% of the infants listed at the participating centers (Fig 1). No information is available on families that did not participate. One boy with a congenital malformation was excluded, leaving 242 infants eligible for participation. Of these, 11 infants were born moderately prematurely (32–36 weeks of gestation) and their age was corrected for prematurity at all assessments. All infants were assessed using SOMP-I by the nurse responsible for the child as part of the regular well-child visits at their respective child health center at as close to 2, 4, 6 and 10 months of age as possible. The nurses noted actions taken such as giving advice regarding motor performance, scheduling an extra follow-up or referring the infant to a pediatrician or physiotherapist.

The participating child health centers represented both urban and rural areas, and the nine nurses in the study were representative of the CHS workforce regarding experience and number of infants in their care. Prior to the study, the nurses underwent a two-day training course led by two pediatric physiotherapists with experience in assessing infants using SOMP-I (KJ and KP). Having completed the course, the nurses began assessing infants using SOMP-I in their clinical practice. The participating nurses were given repeated clinical guidance throughout the study period ranging from questions regarding individual infants to how to address the findings from the assessments, including a brochure with the most common problems and corresponding advice to parents on infant motor performance.

### SOMP-I

SOMP-I measures motor performance in two domains; level of motor development and quality of motor performance at the infant’s own achieved level[60,61]. The assessment is guided by a predefined structure to elicit the infant’s best motor performance during goal-directed activity. The infant is assessed in the supine and prone positions, as well as in sitting, standing and during locomotion. In the supine and prone positions the head, arms and hands, trunk and legs and feet are assessed separately. Hand function is also assessed. Each scale consists of numbered ascending levels (Table 1), and the infant’s achieved level for all 12 scales are added. The maximum total score is 72 points.

**Table 1:** Scale for the evaluation of head movements in the supine position according to the Structured Observation of Motor Performance in Infancy.

Scales		Level of motor development		Quality of motor performance
Supine				
	Head	0	None of the below.	
		1	Head lies to the side.	The infant is not able to control its head movements. The head lies to both sides equally. The head is not laterally flexed or hyperextended.
		2	Keeps head briefly in midline.	The infant keeps its head facing forward for a few seconds. The head falls to both sides equally. The head is not laterally flexed or hyperextended.
		3	Keeps head in midline and starts to turn head.	The infant keeps its head facing forward as long as it wants to, and begins to follow faces/objects/sounds toward both sides equally. The head is not laterally flexed or hyperextended.
		4	Keeps head in midline and turns head completely to the side.	The infant keeps its head stable and facing forward as long as it wants to, and turns its head completely to both sides equally. The head is not laterally flexed or hyperextended.
		5	Moves head freely.	The infant has full control of its head movements, and is able to follow objects with its gaze and head upwards and to the sides without losing balance or triggering involuntary movements in other parts of the body. The head is not rotated, laterally flexed, or hyperextended.

The quality of motor performance is defined at each level of motor development for all scales (Table 1). If the infant performs as expected at the achieved level, the quality is assessed as being adequate (0 points), while a deviation from this definition is scored as being suspected (1 point) or clear (2 points). When more than one deviation is observed, the deviation score is recorded for the most evident of these. The deviation scores for the infant’s achieved levels for all 12 scales are added, creating a maximum total score of 24 points. Note that deviations in this context refer only to deviations from the quality definition for the infant’s achieved level of motor development[60,61].

**Fig 2:**
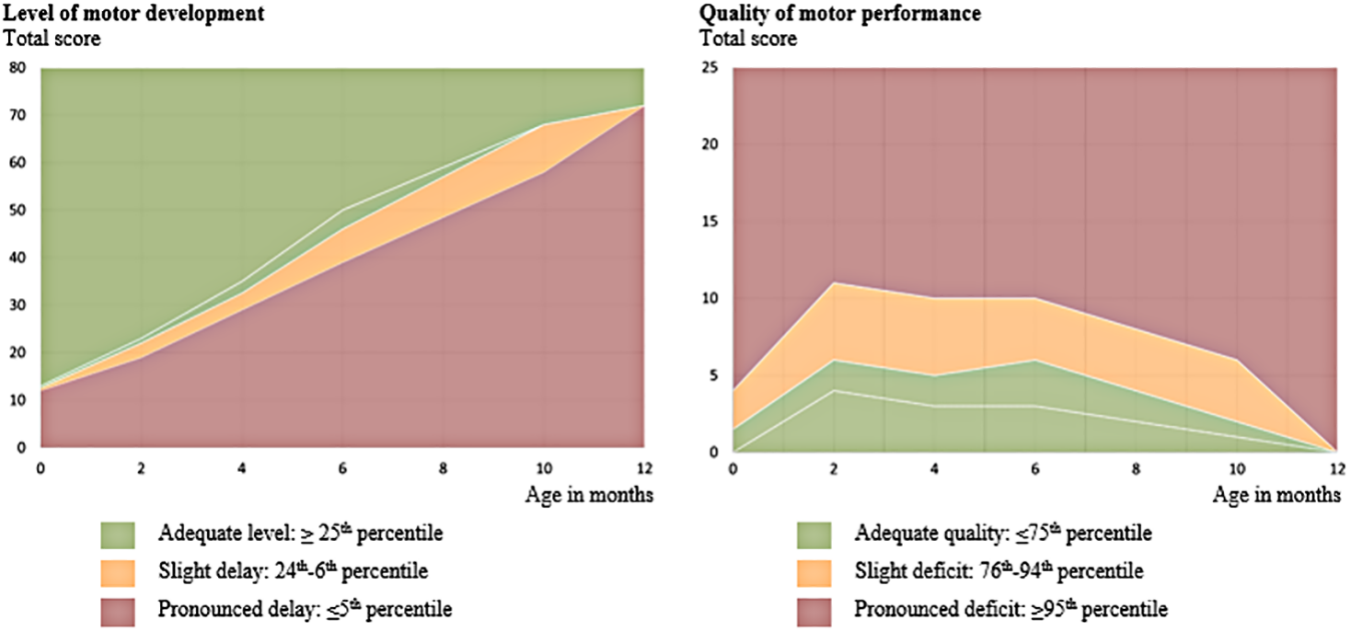
The percentile distribution of Structured Observation of Motor Performance in Infants. Total scores for level and quality are plotted at the infant’s age, corrected for prematurity when appropriate. The percentile distribution was calculated using a reference group of neonatally healthy infants assessed longitudinally using Structured Observation of Motor Performance in Infants (SOMP-I)[59]. The white line in the green area in both graphs represents the 50^th^ percentile. Reprinted from Persson K and Johansen K (2017) Structured Observation of Motor Performance in Infants [60] under a CC BY-NC-ND 4.0 license, with permission from Barnens rörelsebyrå Uppsala ek. för., original copyright 2017.

After the assessment, the total scores for level and quality are plotted within a percentile distribution for each domain at the infant’s age, corrected for prematurity when appropriate (Fig 2). SOMP-I has not yet been normed, instead, a percentile distribution based on longitudinal assessments of 72 neonatally healthy Swedish infants is used as a reference[59]. The three categories in the percentile distribution are based on the distribution of this reference group, where adequate level is at or above the 25^th^ percentile, slight delay ranges between the 6^th^-24^th^ percentile and pronounced delay is at or below the 5^th^ percentile. Conversely, adequate quality is at or below the 75^th^ percentile, slight deficit between the 76^th^-94^th^ percentile and pronounced deficit at or above the 95^th^ percentile.

SOMP-I has shown to be reliable and valid,[59,61,64,65] and detected CP early when used by physiotherapists[66].

### Actions taken by the nurses

Using SOMP-I in the clinical setting is not only an assessment method, but also an aid for intervention. An intervention that can range from giving general advice to stimulate motor development to targeted and tailored advice to specific quality deviations. In example, the assessment revealed that the infant had reached level 3 for the head in supine position (Table 1), but only turned its head to one side. The infant does then not fulfill the criteria for the quality of motor performance at that specific level, and would receive a deviation score of 2 points. An advice could then be to stimulate active rotation of the head towards the side with limited rotation. The majority of advices that was taught to the nurses were active, goal-directed and task specific activities.

No guidelines were given to when to provide advice, scheduling an extra follow-up or referring the infant to specialized care, and this was decided by the nurses if she deemed it was necessary. In the clinic a slight delay in level (≤25^th^ percentile) and/or a slight quality deficit (≥75^th^ percentile) is likely to result in the assessor taking actions.

### Follow-up at 18 months

The children’s motor development was reassessed at 18 months of age through review of their medical records. The gross motor milestone routinely reported in the chart at this age is whether the child walks independently or not. This task is easily and reliably observed by the nurse and failure to attain this milestone may be indicative of major motor problems. We therefore used this measure to ensure that infants with major motor impairments were not missed. If the information could not be retrieved through the medical record, the project leader (KJ) called the parents of the missing children and asked them to report if the child was walking independently at 18 months of age and if there had been any concern regarding the infant’s motor development during the first year of life.

### Ethical approval

The study was approved by the Regional Ethical Review Board in Uppsala, Sweden (dnr 2012/442). Parents provided written informed consent for their infant’s participation and follow-up. Participation was voluntary and withdrawal from the study could take place at any time without consequence.

### Statistical analysis

The percentile distribution in SOMP-I was used to classify the infants’ level of motor development and quality of motor performance. Additionally, the two domains were combined by categorizing the infants in an OR/AND condition, i.e. the infant’s total score(s) fell outside the percentile cut-off(s) in one or both domains, or an AND condition, i.e. the infant’s total scores fell outside the cut-offs for both level and quality.

To analyze the results for the combined conditions, repeated assessments and actions taken, the infants’ motor performance was dichotomized into adequate or delayed level and adequate or deviant quality. The cut-offs were set at slight delay (≤25^th^ percentile) and slight deficit (≥75^th^ percentile), as an outcome outside these cut-offs are likely to result in the assessor taking actions. Descriptive statistics were used when analyzing the outcomes at each assessment, repeated assessments and the actions taken at each assessment age as well as the follow-up at 18 months.

To further analyze the actions taken by the nurses after the SOMP-I assessments, odds ratios (OR) with 95% confidence intervals were calculated using Pearson’s chi-square with the dichotomized outcome of the SOMP-I assessments (adequate or not) as the independent variable and the actions taken (yes or no) by the nurse as the dependent variable. This analysis was performed using the combined conditions and the outcome of repeated assessments. For repeated assessments all adequate assessments were compared to one positive assessment or two or more positive assessments. A significance level of p<0.05 and two-tailed analyses were applied throughout. All calculations were performed using IBM SPSS Statistics 22 for Windows (SPSS Inc., Chicago, IL, USA).

### Imputation for missing data

Two infants had an incomplete SOMP-I assessment in one position, and data were imputed for this level in this position. The total score for the assessed levels was calculated and divided by the number of scales in SOMP-I. This mean score was then substituted for the missing data.

## Results

The nurses performed 936 assessments using SOMP-I within regular practice, and the infants included were assessed at as close to 2 (n = 240), 4 (n = 241), 6 (n = 228) and 10 months (n = 227) as possible, corrected for prematurity when appropriate (Fig 1). The nurses performed on average 82 assessments using SOMP-I (range 49–191). Each assessment took an average of 10 minutes to perform and an additional 5 minutes to score. Of the 242 infants, 87% (n = 210) participated in all four assessments. Another 12% (n = 29) participated in three assessments and 1% (n = 3) participated in two assessments. One of these three infants withdrew from the study after the second assessment. Missed assessments were due to the infant being ill, the family not being able to attend, incomplete documentation or clinical circumstances that precluded the visit. One assessment could not be performed at the 6-month assessment due to lack of cooperation from the infant. This infant had two assessments with results outside the cut-off(s) and was referred to a physiotherapist prior to the 6-month assessment. At 18 months, the children’s motor development was reassessed through review of the medical records (n = 226) or parental report (n = 14). One child was lost to follow-up.

### Outcomes of the SOMP-I assessments

At the 2- and 10-month assessments, approximately 25% of the infants performed outside the cut-off for level of motor development (Table 2). At the 4- and 6-month assessment, less than 5% were assessed as being delayed. Few infants were assessed as having a deficit in their quality of motor performance at each assessment age, and quality deficits were mainly observed during the first months of life. Since the greatest proportion of infants fell outside the cut-off at 2 and 10 months of age this was also when most infants were categorized in the combined conditions (Table 2). The majority of the infants were assessed as adequate in level and quality at all assessments (46%) or delayed in level and/or deficient in quality once (38%) (Table 3). No infants were assessed as having a combined delay and quality deficit more than twice.

**Table 2:** The motor performance of 242 infants presented as number and percentages when assessed by child health nurses using Structured Observation of Motor Performance in Infants.

Months	Percentile category	Level		Quality		Outcome dichotomized	OR/AND		AND	
		n	%	N	%		N	%	N	%
2	Adequate	178	74.2	221	92.1	Adequate	164	68.3	233	97.5
	Slight	38	15.7	17	7.1	Delayed level/	76	31.7	6	2.5
	Pronounced	24	9.9	2	0.8	quality deficit				
4	Adequate	234	97.1	217	90.0	Adequate	212	88.0	240	99.6
	Slight	4	1.7	23	9.5	Delayed level/	29	12.0	1	0.4
	Pronounced	3	1.2	1	0.4	quality deficit				
6	Adequate	214	93.9	217	95.2	Adequate	206	91.2	224	99.1
	Slight	11	4.8	10	4.4	Delayed level/	20	8.8	2	0.9
	Pronounced	2	0.4	1	0.4	quality deficit				
10	Adequate	171	75.3	218	96.0	Adequate	168	74	221	97.4
	Slight	55	24.2	8	3.5	Delayed level/	59	26	6	2.6
	Pronounced	1	0.4	1	0.4	quality deficit				

**Table 3:** Results of repeated assessments using Structured Observation of Motor Performance in Infants.

		Level		Quality		OR/AND		AND	
		n	%	n	%	N	%	N	%
All adequate		136	56.2	194	80.2	110	45.5	226	94.2
Outside cut-off*	Once	81	33.5	35	14.5	91	37.6	13	5.4
	Twice	20	8.3	12	5.0	32	13.2	1	0.4
	Three times	3	1.2	1	0.4	7	2.9		
	Four times	2	0.8			2	0.8		

*Infants with a score outside the percentile cut-off for level, quality or both

### Actions taken by the nurses

Half of the assessments at 2 months of age resulted in some type of action taken by the nurses (Table 4). Although actions were taken at this age even when the infant was assessed as adequate, the infants who had a delay in level and/or a deficit in quality according to SOMP-I were targeted more frequently. This became increasingly evident with age as well as if the infant was assessed as having a combined delay and quality deficit. No information about which advice was given to all infants is available, but advice to increase “tummy-time”, to spend more time on the floor and measures to prevent plagiocephaly was often reported by the nurses.

**Table 4:** Number and percentage of infants for whom additional actions were taken by the nurse with respect to outcome according to Structured Observation of Motor Performance in Infants.

Additional actions taken		2 months		4 months		6 months		10 months		Total	
		n	%	n	%	n	%	n	%	N	%
No		101	45.1	180	84.8	189	84.8	190	85.6	85	35.1
Yes		123	54.9	57	15.2	34	15.2	32	14.4	157	64.9
	Adequate level and quality	62	41	44	21	25	12	7	4	55	50
	OR/AND	61	84	13	46	8	40	24	41	102	77
	AND	5	83	1	100	1	50	4	67	13	93
Missing		18		5		19		20			

When the nurses assessed an infant as being delayed in level and/or deficient in quality (OR/AND), they were three times more likely to take additional actions compared to when infants were assessed as being adequate in both level and quality (OR: 3.4, p<0.001) (Table 5). The likelihood that the nurses took additional actions increased substantially when the infant was assessed as being delayed with a quality deficit (AND; OR: 13.0, p = 0.002) and when the infant repeatedly fell outside the cut-off(s) (OR: 12.7, p<0.001). Even when the nurse assessed the infants as delayed and/or having a quality deficit (OR/AND) once, they were twice as likely to take additional actions compared to when the infant was assessed as adequate in both level and quality at all assessments (OR: 2.4, p-value 0.004).

**Table 5:** The likelihood that additional actions were taken by the child health nurses when an infant performed outside the cut-off for Structured Observation of Motor Performance in Infants.

		OR	95% CI	P-value
All assessments*				
	OR/AND	3.4	2.0, 5.9	.000
	AND	13.0	1.6, 102.8	.002
Longitudinal assessment*				
	All adequate/one positive assessment	2.4	1.3, 4.3	.004
	All adequate/two or more positive assessments	12.7	3.7, 43.5	.000

Note- Pearson’s chi-square analyses presented as odds ratios (OR) with 95% confidence intervals (CI).

*Infants with a score outside the percentile cut-off for level, quality or both were compared to the infants assessed as adequate for both level and quality.

Although the infants that fell outside the cut-off(s) evoked additional actions to a greater extent than infants assessed as adequate, less than half of the infants categorized in the OR/AND condition received additional actions at the 4-, 6- and 10-month assessments (Table 4). The majority of the infants assessed as having a combined delay and quality deficits (AND) were the subject of additional actions.

Eight (1 girl) of the 242 infants (3%) were referred to a physiotherapist due to side differences, plagiocephaly or delayed motor development. All referred infants, except for one where the referral never arrived, were assessed as in need of intervention by the physiotherapist. The intervention started typically around 5 months of age (1–11 months), and the infants had on average four visits (1–11) to the physiotherapist. Of the eight infants referred to a physiotherapist, four had been assessed by the nurses as having both delay with quality deficit at least once (AND) according to SOMP-I, and three of these were also assessed as delayed and/or deficient in quality three times or more (OR/AND). One infant was referred to a pediatrician due to plagiocephaly, but no further information was available.

### The follow-up at 18 months

The follow-up of motor development showed that 98% (n = 236) of the children walked independently by 18 months of age. The four children that did not walk independently were all assessed as slightly delayed in level with an adequate quality of motor performance according to SOMP-I at the 10-month assessment. Two of these infants were diagnosed by a pediatrician as having dissociated motor development, but were not referred to a physiotherapist. No other motor problems were reported in the children’s medical records or by the parents.

## Discussion

Although the nurses in a previous study expressed concern about introducing a more time-consuming assessment in an already tight clinical schedule,[62] our present results suggest that SOMP-I has clinical utility, i.e. is appropriate, practicable and acceptable, when used by nurses in the child healthcare setting. The standardized assessment was integrated in routine care with almost a thousand assessments performed on 242 infants at four time points during the first year of life, and the method appears to have supported the nurses in their clinical-decision making. Nurses are required to perform developmental assessments as a part of the developmental surveillance,[36] and SOMP-I provided the nurses with a tool to master a central task in their daily clinical practice[62].

At 2 and 10 months, the assessment of level of motor development showed a distribution corresponding to the percentile distribution of SOMP-I, suggesting that the nurses’ assessments and the infants in the sample were congruent with those of the original reference population. That 25% of the infants were assessed as delayed in level can have clinical implications. Using a low threshold cut-off could lead to many false-positive results and an over-referral for early intervention, which can be costly for the clinic. On the other hand, King and Glascoe recommend lowering the threshold for referring children with possible developmental problems in order to initiate early intervention[67]. In our study, an outcome outside the cut-off often resulted in the nurses taking actions, indicating that the nurses themselves gave advice and followed-up before referring the infant to specialized care. The ability to give advice and follow-up was previously reported by the nurses to be a great advantage of learning the method[62].

Use of a more stringent cut-off would likely still detect infants with severe motor problems, such as CP,[66] but at the expense of missing larger numbers of infants with mild or moderate motor problems. These motor problems could reasonably be dealt with directly during the child health visit, seen at follow-up or referred for consultation, depending on the type and severity of the identified problem.

Studies have suggested that without using evidence-based assessment methods practitioners may have greater difficulties detecting mild delays or suspected deviations compared to not using an evidence-based assessment method[16,40,68–70]. A Swedish study reported that the average age for referral of children with CP from the CHS to rehabilitation services was 12 months[69]. This is comparable to countries that do not have regular well-child visits, indicating that the developmental surveillance only made a small contribution to early detection of CP. In the above mentioned study only one-third of the infants with mild CP were referred to rehabilitation services during the first year of life[69]. Magnusson and colleagues reported similar findings when evaluating the detection rate for moderate to severe health problems within the CHS. Only 20% of the infants with evident deviations were detected through the regular well-child visits during the first year of life[71]. Consequently, many children with aberrant development, especially mild problems, are denied the opportunity of early beneficial interventions. For these infants, an evidence-based assessment would be a gateway to specialized care and early intervention.

The regular well-child visits and developmental surveillance during the first year of life offer the possibility of repeated assessments, which is known to improve the predictive ability[18,23–26]. A study that investigated the ability of SOMP-I to detect CP showed that the predictive ability for CP increased with repeated assessments,[66] while the results from the present study revealed that infants who showed a delayed level and/or deficient quality at repeated assessments were more likely to receive additional actions in routine care. These findings suggest that performing the structured assessment repeatedly can support the practitioner in clinical decision-making.

That fewer infants than expected were assessed as delayed in level at the 4- and 6-month assessment and as deficient in quality at all assessments may be explained by the nurses’ relative inexperience in observing motor performance in the detail that SOMP-I requires. Given the nurses’ brief exposure to the method as well as differences in educational background and professional expertise compared to physiotherapists, the proficiency they demonstrated as assessors may be seen as quite favorable.

Learning to assess infants using any systematic method is an acquired skill that demands practice over time. Acquiring a skill in the clinical setting should hence not be viewed as merely learning a technical procedure, but rather as a complex process that is influenced by the nurse’s knowledge, experience and intention together with the clinical context itself[72]. In this study, the nurses not only had to learn a new procedure to assess early motor performance, they also had to learn a new way to understand motor development. Quality of motor performance was an entirely new concept for the nurses, and as such was more demanding[62]. We expect that, with time and practical training, nurses who routinely use the SOMP-I method for all infants will become proficient at assessing quality as well. Being able to focus time and knowledge on a specialized field has previously been pointed out as important to develop solid child health competence[73].

More infants were assessed as delayed at 2- and 10-months, which might be explained by the way the level of motor development is constructed. At two months of age, infants have less volitional movements than at 4 and 6 months and may therefore be easier to assess. At 10 months, the infant is expected to sit independently, transfer between positions, crawl or walk, and most infants show an initial pincer grasp. These are skills similar to milestone attainment, and could therefore be familiar for the nurse to observe. Furthermore, few infants in the study population were assessed as having a pronounced delay and/or a pronounced quality deficit, and it is known that the identification and interpretation of subtle anomalies requires more extensive training and experience compared to both normal findings and more severe motor disorders[20,40,69].

The infants that fell outside the cut-off(s) when assessed using SOMP-I were offered additional actions to a greater extent compared to infants assessed as adequate in level and in quality. This is a key finding indicating that using the method supported the nurses in the clinical decision-making and stimulated targeted advice. For instance, interventions initiated before flattening of the skull occurs, i.e. during the first months of life, have shown to be most effective[10]. In our study, half of the assessments at 2 months of age resulted in additional actions, mainly advice to parents to increase “tummy-time” and other measures to prevent plagiocephaly. This suggests that SOMP-I supported the nurses in giving targeted advice in addition to providing anticipatory guidance and general preventive information.

However, not all infants that fell outside the cut-off(s) were provided with additional actions. This may be because the nurses forgot to fill in this information, but they may also have chosen to override the standardized assessment based on information from the general health assessment, previous assessments and/or past medical history. Thomas and colleagues suggested that using informal judgment to override standardized screening results or an inability to interpret the results were the reasons that clinicians did not to adhere to standardized assessment results[16]. This may also reflect a lack of knowledge and experience in giving specific advice regarding motor performance or a reluctance to address problems that might worry the parents. Lennartsson and colleagues found similar results when introducing guidelines to detect plagiocephaly within the CHS, i.e. that nurses did not always give advice when it was indicated[10]. This points to a need for recurring information, training and mentoring as well as follow-up of guidelines when new methods are introduced in clinical practice.

Even though standardized methods are known to detect more children with developmental problems than routine care,[2,10,16–19] this does not automatically lead to earlier referral to specialized services[16]. In our study, no guidelines were provided specifying the actions to take given certain outcomes. Although not reported in the results, there was a difference between the child health centers mainly with regard to extra scheduled follow-ups and referrals to a physiotherapist. This variability could partially be explained by the lack of guidelines for actions to take when an infant fell outside the cut-off(s) as well as unclear referral pathways. If SOMP-I is to be introduced within the CHS, explicit guidelines should be developed to support the child health nurse in decision-making[2,10,16].

Given that early detection could improve outcomes, it is important that the professionals who monitor child development have a method that is able to detect early motor problems without having to rely on parental assessment or parental concern. That the infants referred to the physiotherapist were assessed for the first time at 5 months of age on average could indicate that SOMP-I supported early detection of aberrant motor performance, which again facilitated early referral to specialized care and timely intervention. Furthermore, the follow-up at 18 months did not reveal any infants with major motor impairments, indicating that the nurses did not miss any infants with severe motor impairment when using SOMP-I.

As no infants with major motor impairments were identified through our study, we cannot comment on the method’s ability to detect these infants early when used by nurses in this setting. To further investigate SOMP-I’s ability to accurately detect specific motor problems in a low-risk population, studies targeting these conditions should be performed to ensure the validity of the method for such conditions. For example, if the aim is to investigate SOMP-I’s ability to detect CP in the CHS a larger sample size is needed given the low prevalence of CP.

The infants in our study represented 67% of the infants listed at the child health centers. In particular, one child health center included fewer infants than expected and stopped inclusion prematurely. Consequently, we lack valuable information on one-third of the infants, and we do not have any data on the families that declined participation or why the nurse did not ask certain families to participate. However, of the enrolled infants, the majority participated at 3–4 assessments, indicating that the families that participated found the assessment acceptable. Exploring the parents’ expectations and experiences of the motor assessments in the CHS during infancy would further enhance our understanding of the clinical utility of SOMP-I in this setting.

The follow-up of the children’s motor abilities at 18 months of age did not reveal any major motor impairments. However, assessing attainment of milestones is a rough measure of motor development,[2,7,15,16] and reaching a milestone such as walking independently cannot exclude a motor problem, e.g. asymmetries or coordination problems. A controlled follow-up study comparing one group assessed using SOMP-I, one group assessed using a parental questionnaire and a control group assessed according to current practice of measuring milestone attainment, together with a follow-up of the three groups with a comprehensive motor assessment during the pre-school years would inform us about effective ways of assessing early motor performance in the CHS and if using SOMP-I has incremental value.

Developing new methods is a process of ongoing testing and adjustments to improve the innovation. Doing research on one’s own innovation requires measures to ensure the objectivity and trustworthiness of the results. The first and second authors of this paper (KJ and KP) are the developers and owners of the SOMP-I method. To ensure objectivity, we involved researchers with no prior experience of the method and no personal interest in the method itself.

The use of standardized assessment methods should be an integrated part of developmental surveillance and health monitoring, and the results should be interpreted together with the information retrieved from history taking, the parents’ description of their child, including questions and concerns, and the evaluation of health determinants[21,22,36,39]. Thus, a developmental assessment, in contrast to screening, is not about deciding if a child has a problem or not, but rather to gather information on both the child’s strengths and abilities as well as possible weaknesses. Having accurate knowledge of the child’s developmental process as well as its strengths and weaknesses is pivotal for successful health monitoring and effective counselling[22].

This study represents a first step in testing SOMP-I in the hands of child health nurses. As such, the study design was simple and straightforward. The results, particularly with respect to the assessment of quality, point to the need for long-term educational programs to develop the nurses’ proficiency in this area. This study included a brief introductory course without criteria for certification. More stringency in this respect would be advantageous to better study psychometric properties and clinical utility of the method in the CHS.

## Conclusion

SOMP-I has favorable clinical utility when used by nurses in the CHS, and SOMP-I appears to be appropriate, practicable and acceptable in the child healthcare setting as tested here. The nurses were able to integrate the standardized assessment in routine care, and using the method appears to have supported them in the clinical decision-making process and in providing anticipatory guidance. Larger studies using a controlled methodology are called for to further analyze the clinical utility of SOMP-I when used in the CHS, including a health economic evaluation to analyze possible costs and benefits of implementing the method in routine child healthcare.

If the method is to be implemented within the CHS, education and supervision for nurses is needed, as well as guidelines and referral pathways when aberrant motor performance is detected. In a broader perspective, if detailed motor assessments are to replace milestone attainment in monitoring child development, these concepts should be taught during basic nursing education and built upon in specialty training.

## Supporting information

S1 File Permission to print. (PDF)

S2 File Dataset. (SAV)
