# Supplementary material for: Clinical utility of the Structured Observation of Motor Performance in Infants within the child health services
Source: PLoS One. 2017 Jul 19;12(7):e0181398. doi: 10.1371/journal.pone.0181398 (PMC5517004; doi:10.1371/journal.pone.0181398)
Supplement: S1 File — (PDF) [file pone.0181398.s001.pdf]

BARNENS RÖRELSEBYRÅ
RÖRELSE • UTVECKLING • KUNSKAP

Permission to print

We hereby grant you permission to reprint the percentile distribution of SOMP-I at no charge subject to the following conditions:

1. If any part of the material to be used (for example, figures) has appeared in our publication with credit or acknowledgement to another source, permission must also be sought from that source. If such permission is not obtained then that material may not be included in your publication.
2. Suitable acknowledgment to the source must be made, either as a footnote or in a reference list at the end of your publication.
3. Reproduction of this material is confined to the purpose for which permission is hereby given.
4. This permission is given for publication in PLOS ONE and should not be used for other commercial purposes.
5. PLOS ONE is given the permission to publish the percentile distribution under a CC BY-NC-ND 4.0 license (https://creativecommons.org/licenses/by-nc-nd/4.0/).

Yours sincerely,

Barnens rörelsebyrå

Barnens rörelsebyrå Uppsala ek.för., is an economic association registered in Sweden with corporate identity number 769621-0769, whose registered office is Flottiljgatan 20, 753 37 Uppsala, Sweden.

Barnens rörelsebyrå Uppsala ek. för.
Tel: +46 73 904 29 94 | **E-post**: info@barnensrorelsebyra.se | www.barnensrorelsebyra.se
Postadress: Flottiljgatan 20 | 753 37 Uppsala
Bankgiro: 624-7704 | **BIC**: ESSESESS | **IBAN**: SE6050000000053681047189
Org.nr: 769621-0769 | Innehar F-skattebevis
